# Additional evidence on the efficacy of different Akirin vaccines assessed on *Anopheles arabiensis* (Diptera: *Culicidae*)

**DOI:** 10.1186/s13071-021-04711-8

**Published:** 2021-04-20

**Authors:** Blaženka D. Letinić, Marinela Contreras, Yael Dahan-Moss, Ingrid Linnekugel, José de la Fuente, Lizette L. Koekemoer

**Affiliations:** 1 Wits Research Institute for Malaria, School of Pathology, Faculty of Health Sciences, University of the Witwatersrand, Johannesburg, South Africa; 2 Centre for Emerging Zoonotic and Parasitic Diseases, National Institute for Communicable Diseases of the National Health Laboratory Service, Johannesburg, South Africa; 3 SaBio. Instituto de Investigacion en Recursos Cinegeticos IREC-CSIC-UCLM-JCCM, Ronda de Toledo s/n, 13005 Ciudad Real, Spain; 4 Interdisciplinary Laboratory of Clinical Analysis, Regional Campus of International Excellence Campus Mare Nostrum, Interlab-UMU, University of Murcia, Espinardo, 30100 Murcia, Spain; 5 South African Vaccine Producers Animal Unit, National Institute for Communicable Diseases of the National Health Laboratory Service, Johannesburg, South Africa; 6 Department of Veterinary Pathobiology, Center for Veterinary Health Sciences, Oklahoma State University, Stillwater, OK 74078 USA

**Keywords:** Malaria, Vector control, Akirin, Subolesin, Immunisation, Recombinant proteins

## Abstract

**Background:**

*Anopheles arabiensis* is an opportunistic malaria vector that rests and feeds outdoors, circumventing current indoor vector control methods. Furthermore, this vector will readily feed on both animals and humans. Targeting this vector while feeding on animals can provide an additional intervention for the current vector control activities. Previous results have displayed the efficacy of using Subolesin/Akirin ortholog vaccines for the control of multiple ectoparasite infestations. This made Akirin a potential antigen for vaccine development against *An. arabiensis*.

**Methods:**

The efficacy of three antigens, namely recombinant Akirin from *An. arabiensis,* recombinant Akirin from *Aedes albopictus*, and recombinant Q38 (Akirin/Subolesin chimera) were evaluated as novel interventions for *An. arabiensis* vector control*.* Immunisation trials were conducted based on the concept that mosquitoes feeding on vaccinated balb/c mice would ingest antibodies specific to the target antigen. The antibodies would interact with the target antigen in the arthropod vector, subsequently disrupting its function.

**Results:**

All three antigens successfully reduced *An. arabiensis* survival and reproductive capacities, with a vaccine efficacy of 68–73%.

**Conclusions:**

These results were the first to show that hosts vaccinated with recombinant Akirin vaccines could develop a protective response against this outdoor malaria transmission vector, thus providing a step towards the development of a novel intervention for *An. arabiensis* vector control.

**Graphic Abstract:**

**Supplementary Information:**

The online version contains supplementary material available at 10.1186/s13071-021-04711-8.

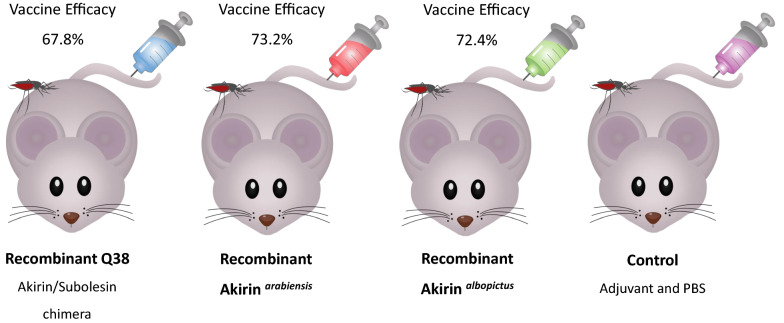

## Background

Traditional control methods for arthropod vectors are mainly based on the use of chemical insecticides [1]. Insecticide resistance, due to increased selection pressure, has necessitated the development of new classes of insecticides or other novel control interventions. Novel approaches that exploit the natural behaviour of arthropod vectors can be used to target these ectoparasites. The opportunistic African malaria vector, *Anopheles arabiensis*, will readily feed on both animals and humans [2]. Targeting this vector while feeding on animals can provide an additional intervention for the current vector control strategies.

Agricultural animals are regularly vaccinated with recombinant vaccines for the control of multiple endo- and ectoparasitic infestations [3–5]. These recombinant vaccines contain protective antigens that develop antigen-specific antibodies in the immunised hosts [6]. Arthropod vectors ingest these antibodies when feeding on the vaccinated hosts. The ingested antibodies interact with and disrupt the function of the target antigen in the arthropod vectors. This results in physiological changes that affect the vector’s biology [7, 8]. This concept was first demonstrated when cattle were vaccinated with crude extracts from *Dermacentor andersoni*. Consequently, these ectoparasites were adversely affected when feeding on the vaccinated hosts [9].

The first arthropod vaccines were manufactured and distributed after the discovery of the gut antigen, BM86, in *Rhipicephalus microplus* [3, 6]. Gavac™ and TickGARD^PLUS^ were used to control tick infestations through cattle immunisation [3, 6, 10–13]. Thereafter, it was proposed that multi-target vaccines directed at the control of several vector species could be developed using evolutionary conserved protective antigens [14]. However, the limiting step in the development of such vaccines is the identification of these antigens [15].

Using expression library immunisation, an evolutionary conserved protective antigen, Subolesin, was discovered in *Ixodes scapularis* [16–18]. Immunisation of cattle, using recombinant Subolesin, caused several deleterious effects in engorged ticks. This included diminished vectorial capacity, reduced oviposition, and reduced vector survivorship [17, 19]. The feeding capabilities of the engorged ticks were also adversely impacted, which resulted in a decline in the vectors’ mass [18]. Additionally, developmental abnormalities were observed. These abnormalities included the failure of nymph metamorphosis as well as tissue damage in the various tick species assessed [17, 20]. Subolesin was discovered to be an ortholog of Akirin in insects after phylogenetic analysis showed a high degree of sequence similarity between these two proteins [21–23].

Akirin is a conserved nuclear transcription co-factor that is involved in multiple biological processes, including innate immunity [21]. Akirin regulates NF-kB-dependent gene expression in the IMD pathway, as it is required at the level of the transcription factor Relish [21, 24–26]. After ingesting a blood meal, the mosquito’s midgut bacterial levels increase rapidly [27]. An increase in the midgut bacterial levels causes an increase in bacterial elicitors, such as peptidoglycan (PGN) [28]. The IMD pathway is activated when PGN binds to a transmembrane peptidoglycan recognition protein known as PGRP-LC [29].

PGN induces a conformational change when binding to PGRP-LC, which causes DREDD to translocate from the nucleus to the plasma membrane [30]. DREDD-mediated cleavage of Relish allows Relish to translocate into the nucleus [31, 32]. In the nucleus, Relish is regulated through interactions with Akirin and the BAP60 component of the Brahma (SWI/SNF) chromatin remodelling complex [21]. This allows for the expression of several antimicrobial peptide-coding genes, such as Attacin and Diptericin [33–35]. The antimicrobial peptides subsequently reduce the number of microbiotas in the mosquito’s midgut to the basal level [33, 34].

The generation of homozygous null Akirin *D. melanogaster* mutants resulted in an embryonic lethal phenotype characteristic of defective muscle patterning, indicating that Akirin also plays a critical role in processes unrelated to immunity, such as embryogenesis [21]. When RNAi-mediated knockdown of *akirin* was performed, a reduction in vector survival and reproductive capacities occurred in several *Culicidae* species [14]. This further displayed the pleiotropic nature of Akirin and made it a potential antigen for vaccine development against various mosquito populations.

Preliminary immunisation studies were evaluated on *Aedes caspius*, *An. atroparvus,* and *Culex pipiens* mosquitoes [14], using immune serum that was acquired from New Zealand white rabbits that were vaccinated with recombinant Akirin from *Ae. albopictus* (Akirin^*albopictus*^)*.* Ingestion of the hyperimmune serum resulted in a decrease of vector survivorship [14], which provided the impetus needed for subsequent recombinant Akirin^*albopictus*^ immunisation studies. Mice were thereafter vaccinated with recombinant Akirin^*albopictus*^ to evaluate the effect of antigen-specific antibodies on *Ae. albopictus* and *An. coluzzii* life parameters [36, 37]. The ingestion of Akirin^*albopictus*^ antibodies diminished reproductive capacities of the vectors; however, it failed to reduced vector survivorship 8 days post-feeding [36, 37]. Another antigen, Q38, was also evaluated on *Ae. albopictus*. The Q38 antigen was a chimera that was designed by combining protective epitopes from *I. scapularis* Subolesin and *Ae. albopictus* Akirin [38]. This antigen was used to evaluate the possibility of using Q38 as a multi-target arthropod vaccine [38]. The ingestion of Q38 antigen-specific antibodies consequently reduced *Ae. albopictus*' reproductive capacities as well as survivorship.

The effects of Akirin immunisation have yet to be evaluated in *An. arabiensis,* which is the main malaria vector in South Africa [39] and other sub-Saharan countries [1]*. Anopheles arabiensis* shows discrepancies in feeding behaviour (endophagic, exophagic) and resting behaviour (endophilic, exophilic) when compared to other *Anopheles* malaria vector species [2]. This behavioural plasticity allows *An. arabiensis* to evade current control methods. Therefore, alternative and additional tools, such as zooprophylaxis, are needed to supplement the current vector control methods to aid in reducing malaria transmission.

RNA-interference mediated knockdown of *akirin* in *An. arabiensis* reduced vector survivorship and reproductive capacities [40], supporting the use of Akirin as a potential antigen for vaccine development against this malaria vector. To determine whether Akirin could represent a novel target for the control of *An. arabiensis*, the efficacy of Akirin^*albopictus*^ [36] and recombinant Q38 [38] was evaluated on *An. arabiensis* life parameters. However, *Aedes* are vectors of diseases such as dengue fever, yellow fever, chikungunya, and Zika virus, among many others, which are unrelated to malaria. Therefore, an additional species-specific recombinant antigen was also evaluated on *An. arabiensis*, namely recombinant Akirin from *An. arabiensis* (Akirin^*arabiensis*^). This was the first time to our knowledge that a species-specific recombinant protein has been evaluated for the genus *Anopheles*.

All three recombinants were evaluated on *An. arabiensis* concurrently to evaluate whether a species-specific vaccine will be advantageous. When evaluated, all three antigens successfully reduced *An. arabiensis* survival and reproductive capacities, with a vaccine efficacy of 68–73%. This highlighted that the immunisation of hosts, using these protective antigens, could be further assessed to aid as a supplementary tool for *An. arabiensis* vector control.

## Methods

### Biological material

A laboratory strain of *An. arabiensis* mosquitoes (MBN) was used. This strain was originally colonised from wild material collected in Mamfene, northern KwaZulu-Natal, South Africa, in 2002 [40, 41].

The colony is maintained under conditions of 80% (± 5%) humidity, 25 °C (± 2 °C), and a 12-h day/night cycle with 45-min dusk/dawn transitions in the Botha De Meillon Insectary, at the Vector Control Reference Laboratory (VCRL) of the National Institute for Communicable Diseases (NICD) in Johannesburg [40, 42]. All adult mosquitoes were sustained on a 10% sucrose solution diet.

Two blood meals were provided to mated female mosquitoes, 5 days apart, from immunised balb/c mice. The balb/c colony was sourced from and maintained at the South African Vaccine Producers (SAVP) animal unit of the NICD, at a temperature range of 21 °C (± 2 °C), with a 12-h day/night cycle. Animal handling, injections, and veterinary procedures were strictly conducted by trained South African Veterinary Council (SAVC) registered staff. The mice did not suffer any ill effects during this study.

### Design and produced recombinant Akirin vaccines

RNA was extracted from 1-day-old female *An. arabiensis* mosquitoes (60 mg), using Tri Reagent (Sigma-Aldrich, T9424), and purified using the RNeasy® MinElute™ clean-up kit (Qiagen, 74204). A NanoDrop™ spectrophotometer was used to assess the concentration of the purified RNA, and the integrity of the RNA was assessed using a 1% non-denaturing agarose gel in TBE (Tris/Borate/EDTA), which was electrophoresed for 30 min at 70 V.

The access RT-PCR system (Promega, A1250) was used to reverse-transcribe RNA into cDNA and amplify the coding region of Akirin (GenBank: KU973613). The forward primer (5′CACCATGGCGTGCGCAACGTTA3′) was used to facilitate directional cloning in the pET TOPO® vector (Invitrogen, K101-01), while the reverse primer (5′GGAGAGGTAACTTGGCGCTGCTTC3′) allowed the PCR product to be cloned in frame with the V5 epitope and the 6 × His-tag in the pET101/D-TOPO® vector (Invitrogen, K101-01). The RT-PCR product was purified using the MinElute® PCR purification kit (Qiagen, 28004), quantified using the NanoDrop™ spectrophotometer, assessed using a 1% agarose gel in TAE (Tris/Acetate/EDTA), and sent for sequencing (Secugen SL, Madrid, Spain).

The purified RT-PCR product was cloned into the pET101/D-TOPO® vector (Invitrogen, K101-01), after which the pET TOPO® construct was transformed into One Shot® TOP10 chemically competent *Escherichia coli* cells (Invitrogen, C6010). The transformed cells were spread on Lysogeny broth (LB) agar plates, containing 50 μg/ml ampicillin. After overnight incubation (37 °C), the GenJet™ plasmid miniprep kit (Invitrogen, K0502) was used to isolate and purify the plasmid DNA from selected colonies. Purified plasmids were quantified using the NanoDrop™ spectrophotometer and sent for sequencing.

The selected, purified plasmid was transformed into recombinant *E. coli* BL21 Star™ (DE3) One Shot® cells (Invitrogen, C6010-03). The transformation reaction was inoculated in LB media, containing 50 μg/ml ampicillin and 0.5% glucose (CONDA, 21528). After an overnight incubation (37 °C, 200 rpm), the *E. coli* cells, containing the plasmid with the recombinant Akirin^*arabiensis*^, were added to a 50% glycerol solution (1:1) and stored at −80 °C, at the Instituto de Investigación en Recursos Cinegéticos, IREC, Ciudad Real, Spain, for future use.

Glycerol stocks containing recombinant Akirin^*arabiensis*^, recombinant Akirin^*albopictus*^ (GenBank: KU973617.1) [14], and recombinant Q38 (GeneBank: JX193856.1) [38] plasmids were expressed using the *E. coli* expression system [43]. The glycerol stocks were inoculated into LB media containing 50 μg/ml ampicillin and 0.5% glucose and were propagated overnight (37 °C, 200 rpm) to a final OD_600nm_ of 0.4. Isopropyl-β-d-1-thiogalactopyranoside (IPTG) (Sigma-Aldrich, 367-93-1) was added to the propagated culture to a final concentration of 0.5 mM. The culture was incubated for an additional 5 h (37 °C, 200 rpm) to induce heterologous protein expression (OD_600nm_ = 1). Centrifugation was conducted to harvest the bacterial cells (3900×*g*, 15 min, 4 °C).

Harvested cells were resuspended in lysis buffer (50 mM potassium phosphate, pH 7.8, 400 mM NaCl, 100 mM KCl, 7 M urea, 10 mM imidazole), which contained a protease inhibitor (Roche, 04693132001). The resuspended cells were mechanically disrupted by sonication (30% amplitude, 30-s on/off pulses) using the Ultrasonic Homogenizer (Bandelin Sonopuls, Model MS73). After sonification, centrifugation was conducted (15,000×*g*, 15 min, 4 °C). The supernatant containing the soluble recombinant protein was purified by nickel affinity chromatography. A 1-ml HisTrap™ FF column (GE Healthcare, 11-0012-38 AH) was mounted on the ÄKTA-FPLC system (GE Healthcare, ÄKTA prime plus), where fractions were eluted in elution buffer (50 mM KH_2_PO_4_ pH 7.8, 400 mM NaCl, 100 mM KCl, 7 M urea, 500 mM imidazole). Eluted fractions, containing the purified protein, were transferred into a cellulose dialysis tubing membrane (Sigma-Aldrich, 3110), and incubated in 1 × PBS (137 mM NaCl, 2.7 mM KCl, 10 mM Na_2_HPO_4_, 1.8 mM KH_2_PO_4_ pH 7.4) for 12 h at 4 °C.

The purified recombinant proteins were quantified using the Pierce™ BCA protein assay kit (ThermoFisher Scientific, 23225), where the total protein quantification was determined using a plate reader (562 nm). A sodium dodecyl sulphate polyacrylamide gel electrophoresis (SDS-PAGE) was used to assess the integrity of the proteins. For each recombinant protein, 10 µg of total protein was loaded on the 12% SDS-PAGE precast gel (C.B.S. Scientific, BK01212-10) with the Spectra™ multicolour broad range protein ladder (ThermoFisher Scientific, 26634) and was electrophoresed (1 h, 180 mA). The gel was either stained with Blue BANDit™ (VWR Life Science, K217) for 1 h or was used for Western blot analysis. After staining, the SDS-PAGE gel was washed overnight in distilled water and visualized using the gel doc system.

For Western blot analysis, the SDS-PAGE gel was transferred to a nitrocellulose membrane. The membrane was blocked with 5% BSA for 2 h at room temperature, washed four times with Tris-buffered saline (TBS; 50 mM Tris–Cl, pH 7.5, 150 mM NaCl, 0.5% Tween 20), and incubated with anti-His_6_ from mouse IgG_1_ (Sigma-Aldrich, 11922416001) at a 1:500 dilution in TBS. The membrane was incubated overnight at 4 °C. Thereafter, the membrane was washed four times with TBS and incubated with an anti-mouse IgG-horseradish peroxidase (HRP) conjugate (Sigma-Aldrich, AP308P) that was diluted to 1:1000 in TBS with 3% BSA. The membrane was washed five times with TBS and finally developed with tetramethylbenzidine (TMB) stabilised substrate for HRP (Promega, W4121), according to the manufacturer's recommendations.

### Mouse immunisation

The recombinant proteins were concentrated to a final concentration of 250 µg/ml in PBS and emulsified with 1.5 ml of adjuvant (Montanide™, ISA 50 V2) to formulate the recombinant vaccines. A vaccine consisting of PBS and adjuvant was used as a negative control. Five-week-old balb/c mice were vaccinated intraperitoneally, using a double-blind approach, where each treatment was given a code and the identity of the code was not revealed to the researchers until the end of the experiment. Therefore, mice were either vaccinated with recombinant protein (25 µg/dose) or the control consisting of PBS and adjuvant (1 mouse per treatment, 4 treatments, 3 replicates, *n* = 12 mice). Each mouse received three doses of vaccine, which were administered 2 weeks apart.

Two weeks after the final vaccination, the immunised mice were used to provide female *An. arabiensis* mosquitoes with two subsequent blood meals. This was done because the mosquito colony was routinely provided with two blood meals before ovipositioning. Therefore, the female mosquitoes were 5 days old at the first feeding and 9 days old at the second feeding. Prior to blood-feeding, female mosquitoes were mated with male mosquitoes over 4 days, using a male to female ratio of 1:1, after which the male mosquitoes were removed from the cages.

Histamine present in the mosquito’s salivary glands did not induce an itch-associated response in mice; thus, the mice did not experience discomfort after blood-feeding [44]. However, to prevent discomfort during feeding, the mice were anaesthetised with a mix of Xylazine and Anaket (0.02–0.03 ml). Once anaesthetised, each mouse was placed on top of their respective mosquito cage (BugDorm, 4M1515). Each cage retained a total of 100 mated female mosquitoes, which were starved 12 h before feeding. The mosquitoes fed for a total of 35 min, after which the mice were removed from the top of each cage. The mosquitoes had a feeding success of approximately 80% per feeding, which was a 66.3% overall feeding success per treatment (2 feedings, 3 replicates, *n* = 199 mosquitoes). Mosquitoes that did not take a blood meal were subsequently removed from the study after feeding; thus, both fully and partially fed mosquitoes were included in the analysis of this study. This would simulate similar results to those of a field setting.

### Mosquito life table parameters

The rate of survival was monitored until 100% mortality was reached for all four treatments, after which a Kaplan-Meier survival curve was constructed using Statistix 10. A log-rank test was performed to determine whether there was a statistically significant difference in rate of survival between the treatments (*p* < 0.05) [40]. Simultaneously, vector fecundity and vector fertility were also analysed. Two days after receiving the second blood meal, filter paper egg plates were placed at the bottom of each cage. The eggs laid on each plate were counted daily, over 5 days. Once the eggs were counted, they were placed into separate plastic containers, filled with 500 ml of dH_2_O (L: 226 mm, × W: 166 mm × H: 10 mm). The hatchlings were counted over 14 days. Vector fecundity was defined by determining the mean number of eggs laid per female mosquito, and vector fertility was defined by determining the mean percentage of hatchlings. A one-way ANOVA test and a Tukey-HSD all pairwise comparison test were used to compare the mean fecundity and fertility between the treatments (*p* < 0.05) [40].

Vaccine efficiency was used to interpret the overall effect of each antigen on the *An. arabiensis* population tested. Vaccine efficiency is a good method to compare the effect of different antigens on a vector population; however, this parameter cannot be compared between vector species, as their developmental processes may differ [5]. Efficiency (E) was calculated considering the effect of vaccination on the reduction of the mean vector survival rate (S), vector fecundity (Fc), and vector fertility (Fr) as E (%) = 100[(1−S/100)(1−Fc/100)(1−Fr/100)] [16]. A one-way ANOVA test and Tukey-HSD all pairwise comparison test were used to compare the vaccine efficacy between the treatments (*p* < 0.05).

### Sample collection

Blood samples were collected from the saphenous vein [45] of each mouse 24 h before each immunisation and blood-feeding. The vein was punctured using a 27.5-gauge needle, where drops of blood were collected. Centrifugation was conducted on the blood samples to separate the serum from the red blood cells (1500×*g*, 10 min, 4 °C). Thereafter, the serum was used to perform an indirect ELISA to determine the antibody titres within the mice. Simultaneously, three engorged mosquitoes were randomly selected from each treatment 24 h after the final blood-feeding (3 mosquitoes per replicate, 3 replicates, *n* = 9 mosquitoes per treatment). These mosquitoes were used to determine whether antibodies from the vaccinated mice were ingested by the engorged females and whether these antibodies could subsequently be detected when performing an indirect ELISA.

### Antibody detection

Each recombinant protein was diluted in 100 μl Tris-buffered saline (25 mM Tris HCl, 150 mM NaCl, 2 mM KCl) (TBS) and used to coat a separate 96-well microplate (0.1 μg). The recombinant proteins were immobilised to the microplate by overnight incubation (4 °C). Thereafter, the microplates were blocked with Blocker™ BLOTTO (ThermoFisher Scientific, 37530) for 2 h at 37 °C. The blocking solution was aspirated off the microplate. The immobilised recombinant proteins were paired to the respective immunogen for the determination of the respective antibody titres, where each treatment was assessed in duplicate. Samples consisting of TBS were used as background controls.

The mouse sera samples or the TBS control samples were diluted in blocking solution (1:20, v/v) and hybridised to the microplate (2 h, 37 °C) to quantify the antibody titres within the mice. For the quantification of antibodies ingested by the mosquitoes, the blood-fed mosquitoes were homogenized in 200 μl TBS-Tween 20 (TBS-T) and centrifuged to collect the supernatant (3 min, 16,000×*g*, 4 °C). The supernatant was subsequently hybridised to the microplate (2 h, 37 °C). After hybridisation, the microplate was washed three times with TBS-T. Goat anti-mouse IgG antibody, conjugated with horseradish peroxidase (HRP) (Agilent, P0447), was diluted in blocking buffer (1:3000). The diluted secondary antibody was bound to the primary antibody through a 2-h incubation (37 °C). Unbound secondary antibody was aspirated off the microplate. The microplate was then washed three times with TBS-T before adding the SigmaFast™ enzyme-substrate (Sigma-Aldrich, N1891).

After a 30-min incubation period, the microplate was analysed at 450 nm using the multi-well plate reader. The antibody titres were represented as OD _450 nm_ (OD _mouse serum or mosquito sample_ − OD _TBS control_). The antibody titres were compared between the control and treated groups, using an unpaired *t* test (*p* < 0.05). Additionally, the fold change of the antibody titres was calculated between the treatments and control group using the formula “(B-A)/A”. A Pearson correlation analysis was also conducted in Microsoft Excel to compare the significant effects of antibody response on *An. arabiensis* lifetable parameters (*r* < −0.5).

## Results

### Mouse antibody detection

**Fig. 1:**
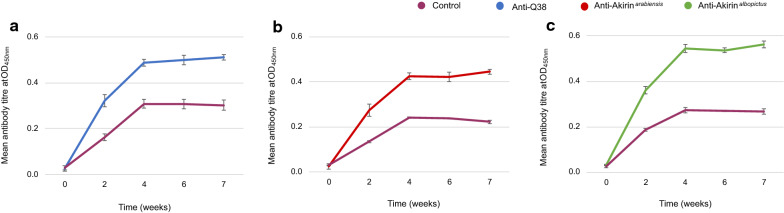
The antibody response in mice vaccinated with recombinant Akirin proteins, assessed 24 h before each immunisation (weeks 0, 2, and 4) and blood-feeding (weeks 6 and 7), at an optical density of 450 nm. The antibody titres of the mice that were vaccinated with recombinant Q38 (blue), recombinant Akirin^*arabiensis*^ (red), and recombinant Akirin^*albopictus*^ (green) proteins were significantly higher than those of the mice vaccinated with the control treatment (purple). The error bars on the graph are indicative of the SE between the treatment replicates (3 mice per treatment, 3 treatments, *n* = 12)

The recombinant proteins were successfully produced using the *E. coli* expression system (Additional file 1), emulsified with the adjuvant to formulate the recombinant vaccines, and administered to the balb/c mice. The antibody titres of the mice that were vaccinated with the recombinant proteins were significantly higher than those of the control mice. The mean antibody titres of the mice that were vaccinated with the recombinant Q38 protein were 0.7-fold higher than the mean antibody titres of the control mice (unpaired *t* test: *t* = 2.46, *p* = 0.05, DF = 1) (Fig. 1a). Similarly, the antibody titres of the mice vaccinated with the recombinant Akirin^*arabiensis*^ protein were 0.9-fold higher than those of the control mice (unpaired *t* test: *t* = 2.98, *p* = 0.03, DF = 1) (Fig. 1b), and the antibody titres of the mice vaccinated with the recombinant Akirin^*albopictus*^ protein were 0.5-fold higher than those of the control mice overall (unpaired *t* test: *t* = 2.90, *p* = 0.03, DF = 1) (Fig. 1c).

### Mosquito antibody detection

The female *An. arabiensis* mosquitoes successfully ingested the antibodies from the vaccinated mice. The antibody titres of the females that ingested blood from the mice vaccinated with recombinant protein were significantly higher than the antibody titres of females that ingested blood from the control mice (3 mosquitoes per replicate, 3 replicates, *n* = 9 mosquitoes per treatment). When the antibodies titres were quantified, it was seen that the *An. arabiensis* females who ingested the anti-Q38 antibodies had an antibody titre that was 1.5-fold higher than the females who ingested blood from the control mice (unpaired *t* test: *t* = 3.25, *p* = 0.03, DF = 1). The *An. arabiensis* females who ingested the anti-Akirin^*arabiensis*^ antibodies had an antibody titre that was 2.6-fold higher than the females who ingested blood from the control mice (unpaired *t* test: *t* = 7.04, *p* < 0.002, DF = 1), and the *An. arabiensis* females who ingested the anti-Akirin^*albopictus*^ antibodies had an antibody titre that was 8.5-fold higher than the females who ingested blood from the control mice (unpaired *t* test: *t* = 11.92, *p* < 0.001, DF = 1).

### Mosquito lifetable parameters

**Fig. 2:**
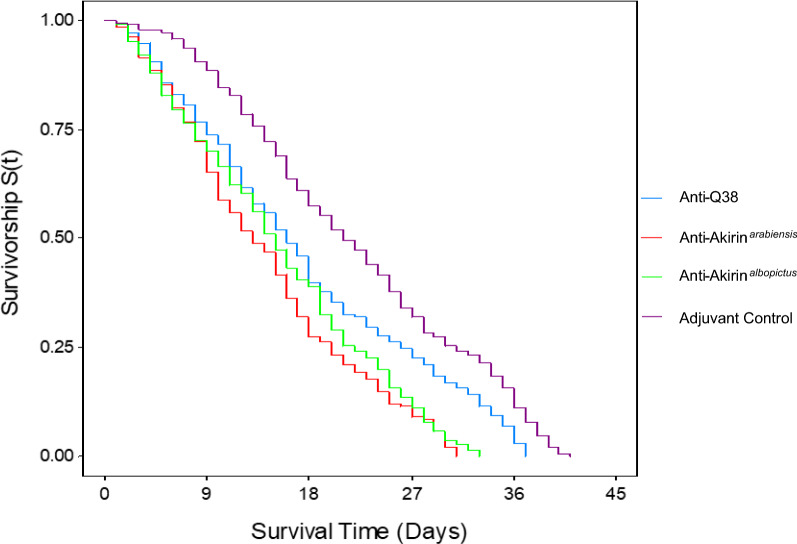
Kaplan-Meier survival analysis of the female *An. arabiensis* mosquitoes assessed post-blood-feeding (190 mosquitoes per treatment). The females that ingested anti-Q38 antibodies (blue), anti-Akirin^*albopictus*^ antibodies (green), and anti- Akirin^*arabiensis*^ antibodies (red) reached 100% mortality 4, 8, and 10 days (respectively) before the females that ingested blood from the control mice (purple). Vector survival was significantly reduced in the females that ingested blood from the mice vaccinated with recombinant proteins compared to the females that ingested blood from the control mice (log-rank: χ^2^ = 84, *p* < 0.01, DF = 3)

Vector survival was significantly reduced in *An. arabiensis* females that ingested blood from the mice that were vaccinated with recombinant proteins compared to the females that ingested blood from the control mice (log-rank: χ^2^ = 84, *p* < 0.01, DF = 3) (Additional file 2). *Anopheles arabiensis* that ingested blood from the control mice had a mean survival time of 21 days, while the females that ingested anti-Q38, anti-Akirin^*albopictus*^, and anti-Akirin^*arabiensis*^ antibodies had a mean survival time of 16, 14, and 13 days respectively. This was a 24%, 33%, and 38% reduction in mean survival time compared to the control treatment (Fig. 2).

**Fig. 3:**
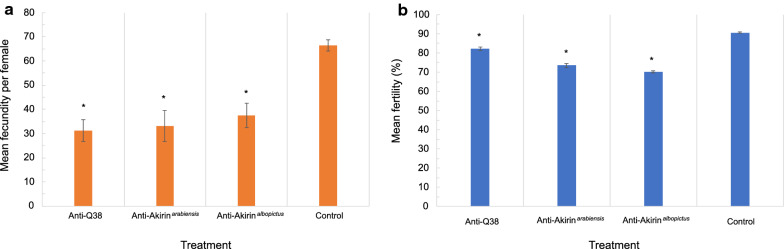
The mean vector fecundity (**a**) and vector fertility (**b**) assessed per female *An. arabiensis* mosquito (*n* = 190 per treatment), post-blood-feeding. Vector fecundity (one-way ANOVA: *p* < 0.001, F = 11.7, DF = 3) and vector fertility (one-way ANOVA: *p* < 0.001, F = 68.9, DF = 3) were significantly reduced in *An. arabiensis* females that ingested antibodies from the mice that were vaccinated with the recombinant proteins compared to the control treatment. The asterisk shows a significant change from the control treatment, while the error bars on the graph are indicative of the SE between the treatment replicates (Additional file 3)

Vector fecundity was also significantly reduced in the *An. arabiensis* females that ingested blood from the mice vaccinated with recombinant proteins compared to the females that ingested blood from the control mice (one-way ANOVA: *p* < 0.001, *F* = 11.7, DF = 3). *Anopheles arabiensis* that ingested blood from the control mice had a mean fecundity of 66 eggs laid per female mosquito, while the females that ingested anti-Q38, anti-Akirin^*albopictus*^, and anti-Akirin^*arabiensis*^ antibodies had a mean fecundity of 31, 38, and 33 eggs laid per female, respectively. This was a 53%, 42%, and 50% reduction in mean fecundity compared to the control treatment (Fig. 3).

Similarly, vector fertility was also significantly reduced in the *An. arabiensis* females that ingested blood from the mice vaccinated with the recombinant proteins compared to the females that ingested blood from the control mice (one-way ANOVA: *p* < 0.001, F = 68.9, DF = 3). *Anopheles arabiensis* that ingested blood from the control mice had a 91% overall hatch rate of the eggs that were laid per female mosquito, while the females that ingested anti-Q38, anti-Akirin^*albopictus*^, and anti-Akirin^*arabiensis*^ antibodies had an overall hatch rate of 82%, 73%, and 70%, respectively. This was a 10%, 23%, and 20% reduction in mean fertility compared to the control treatment (Fig. 3).

When comparing the lifetable parameters between the various treatments, it was seen that the *An. arabiensis* populations that ingested the antibodies from the mice vaccinated with recombinant proteins were adversely affected compared to the *An. arabiensis* mosquitoes that ingested blood from the control mice (Table 1). Overall, the recombinant Q38 antigen had a vaccine efficacy of 67.8% on the targeted *An. arabiensis* vector population, while the recombinant Akirin^*albopictus*^ antigen had a vaccine efficacy of 72.4%, and the recombinant Akirin^*arabiensis*^ antigen had a vaccine efficacy of 73.2% (Additional file 4).

**Table 1:** A comparison of the lifetable parameters between the *An. arabiensis* female mosquitoes that ingested blood from the control mice and the *An. arabiensis* females that ingested blood from the mice that were vaccinated with recombinant Q38, Akirin^*arabiensis*^, and Akirin^*albopictus*^ proteins

Treatment	Control	Q38	Akirin^*arabiensis*^	Akirin^*albopictus*^
Mean survival rate (days) ± SD; N	22	14	12	15
	21	15	12	14
	20	18	15	15
	(21 ± 1; 190) ^a^	(16 ± 2; 190) ^b^	(13 ± 2; 190) ^b^	(14 ± 1; 190) ^b^
Fecundity (eggs laid/ total amount of females) ± SD; N	68	37	29	28
	70	22	25	40
	62	34	46	45
	(66 ± 4; 190) ^a^	(31 ± 8; 190) ^b^	(33 ± 11; 190) ^b^	38 ± 9; 190) ^b^
Fertility (eggs hatched/eggs laid) ± SD; N	0.9	0.8	0.7	0.7
	0.9	0.8	0.7	0.7
	0.9	0.8	0.8	0.7
	(0.9 ± 0.1; 190) ^a^	(0.8 ± 0.1; 190) ^b^	(0.7 ± 0.2; 190) ^c^	0.7 ± 0.1; 190) ^c^
Vaccine efficacy (%)	-	67.8 ^a^	73.2 ^a^	72.4 ^a^

*The results are shown for each vaccine trial (*n* = 3 replicates), with the average standard deviation (± SD) and the total sample size (N) assessed. The data were analysed using either the log-rank test (*p* < 0.05) or a one-way ANOVA test (*p* < 0.05). The letters “a–c” were used to represent statistical differences between the various groups when conducting the Tukey-HSD test

**Fig. 4:**
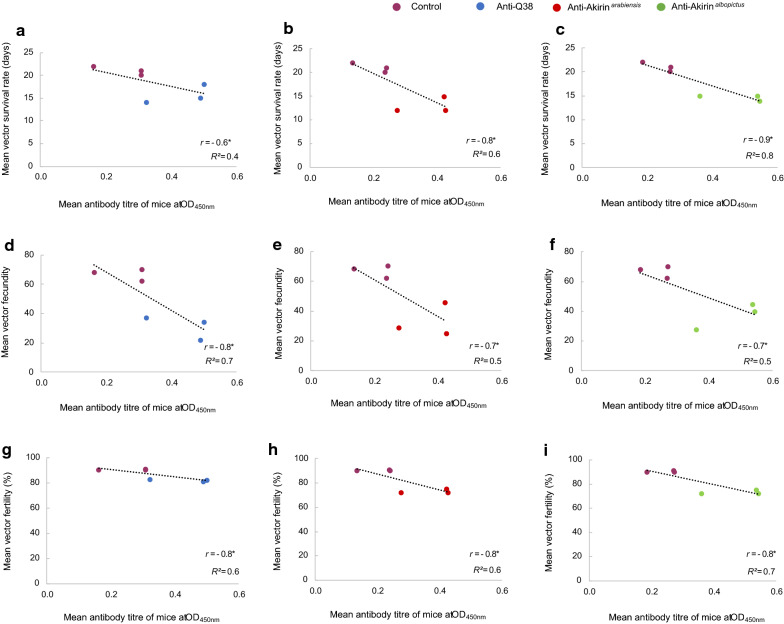
The effect of the antibody response of the vaccinated mice on *An. arabiensis* lifetable parameters post-blood-feeding. The antibody titres of the mice negatively correlated with the effects observed on the vector survival (**a**–**c**), vector fecundity (**d**–**f**), and vector fertility (**g**–**i**) of the *An. arabiensis* populations that ingested the anti-Q38 antibodies (blue), anti-Akirin^*arabiensis*^ antibodies (red), and anti-Akirin^*albopictus*^ antibodies (green). The linear correlation coefficients (*R*^2^) and Pearson correlation coefficient are shown (*r < -0.5; *n* = 6)

The adverse effects observed on the *An. arabiensis* populations that ingested the anti-Q38, anti-Akirin^*arabiensis*^*,* and anti-Akirin^*albopictus*^ antibodies negatively correlated with the mean antibody titres of the vaccinated mice (Fig. 4).

## Discussion

The significant reduction in vector survival and reproductive capacities characterised by the knockdown of *akirin* in *An. arabiensis* [40] made Akirin a potential antigen for vaccine development against the untargeted outdoor vector populations. Thus, to determine whether Akirin could represent a novel target for the control of *An. arabiensis*, the efficacy of three recombinant vaccines was evaluated concurrently, namely recombinant Akirin^*arabiensis*^, recombinant Akirin^*albopictus*^, and recombinant Q38. Antibodies against the recombinant proteins were effectively elicited in vaccinated mice and were successfully ingested by the engorged *An. arabiensis.* Thereafter, vector lifetable parameters such as fertility, fecundity, and longevity were assessed.

*Anopheles arabiensis* longevity was significantly reduced when anti-Akirin^*arabiensis*^ antibodies, anti- Akirin^*albopictus*^ antibodies, and anti-Q38 antibodies were ingested. Although not completely understood, it is thought that the ingested antibodies cross the cell membrane and interact with the target protein in the cytoplasm, thus preventing Akirin from translocation to the nucleus [8, 46]. Previous Akirin gene regulation analysis, performed in *An. coluzzii*, showed that *akirin* expression was downregulated by 16–40% in the mosquito midgut and 25–65% in the remaining tissue [37]. This shows that *akirin* is expressed constitutively through the mosquito’s body and thus explains the profound effects observed on the vector’s biology when Akirin is targeted.

As aforementioned, Akirin regulates NF-kB dependent gene expression in the IMD pathway, which allows for the gene expression of several antimicrobial peptide-coding genes [21, 25, 26, 33–35]. Since the antimicrobial peptides reduce the number of microbiotas in the midgut to the basal level [33, 34], it is possible that targeting Akirin disrupts bacterial homeostasis in *An. arabiensis.* Disrupting the bacterial homeostasis would weaken the innate immune defence in *An. arabiensis,* which would ultimately affect the survival of the vector [21, 27, 47]. However, additional research is needed to verify this hypothesis.

Mosquito longevity is a critical factor regarding malaria transmission [48]. To transmit malaria, a female *Anopheles* mosquito must ingest a blood meal infected with gametocytes. The gametocytes must encounter the midgut epithelium, undergo fertilization, develop into motile ookinetes, traverse across the midgut epithelium, develop into oocysts, produce haploid sporozoites, and migrate through the mosquito haemolymph to the salivary glands to complete the sporogonic cycle [49, 50]. It takes approximately 14 days to complete this cycle. Once the sporogonic cycle is complete, the sporozoites are injected into the next vertebrate host when taking a successive blood meal [51, 52]. This allows for the transmission of malaria. When the female *An. arabiensis* mosquitoes ingested the anti-Q38 antibodies, anti-Akirin^*albopictus*^ antibodies, and anti- Akirin^*arabiensis*^ antibodies, they had a mean survival time of 16, 14, and 13 days, respectively. Eliminating a third of the vector population before the completion of the sporogonic cycle could significantly affect the transmission of malaria.

Additionally, both vector fecundity and vector fertility were significantly reduced when anti-Akirin^*arabiensis*^ antibodies, anti-Akirin^*albopictus*^ antibodies, and anti-Q38 antibodies were ingested by *An. arabiensis*. It has been postulated that the movement of antibodies to reproductive tissues interferes with oogenesis, as the synthesis of vitellogenin by the fat bodies as well as its uptake of vitellogenin from the haemolymph may be inhibited [53]. Alternatively, it has been suggested that the ingested mosquito antibodies may irritate the gut, which in turn may reduce the uptake of a blood meal or the availability of nutrients. The content of some of the developing follicles may therefore be reabsorbed, resulting in reduced vector ovipositioning [53]. This, however, would need further investigation. The impact on larval development were not investigated in this study, but future studies should take this into consideration.

Akirin, however, is known to play a critical role in processes unrelated to immunity, such as development and reproduction [14, 21]. As aforementioned, immunisation of cattle, using the recombinant ortholog, Subolesin, had several deleterious effects on the engorged ticks. This included developmental abnormalities, such as the failure of nymph metamorphosis and tissue damage in the various tick species assessed, as well as diminished vector ovipositioning [17, 19, 20]. Regardless of the underlying mechanism, reducing the number of *An. arabiensis* eggs laid by half and the amount of viable *An. arabiensis* progeny by a fifth could decrease the vector population. It will be vital to investigate the impact of the recombinant vaccine on vector susceptibility to the human *Plasmodium* parasite.

In this study, the female *An. arabiensis* mosquitoes were allowed to feed on each of the respective mice for a total of 35 min. As aforementioned the original concept of concealed antigen identification and vaccination originated in ticks. Ticks are long-duration feeders, whereas mosquitoes are short-duration feeders. Mosquitoes, therefore, ingest less blood than ticks. However, mosquitoes ingest an additional blood meal every 2 to 3 days. This would ensure repeated ingestion of the anti-mosquito antibodies, which could disrupt gut bacterial homeostasis, consequently reducing the lifespan of the vector [27] as well as its reproductive fitness. Reducing both *An. arabiensis* survivorship and reproductive fitness would ultimately cause a reduction in the vector’s population abundance. A reduction in vector abundance would have a significant impact on malaria transmission.

It should be noted that the adverse effects that were observed on the *An. arabiensis* population that ingested the anti-Akirin^*albopictus*^ antibodies was an interesting observation. Previous recombinant Akirin^*albopictus*^ immunisation experiments affected *Ae. aegypti*, *Ae. albopictus,* and *An. coluzzii* oviposition; however, the ingestion of anti-Akirin^*albopictus*^ antibodies did not affect vector longevity [36, 37]. These differences can be attributed to various experimental factors that need further investigation. The sample size that was used to assess the effect of anti-Akirin vaccines on *An. arabiensis* was much larger than those described in past studies [36, 37]. Additionally, the previous studies only provided a single blood meal to the female mosquitoes [36, 37], while *An. arabiensis* were provided with two blood meals. The repeated ingestion of the anti-mosquito antibodies could have added to the adverse effects observed on the mosquito lifetable parameters. Furthermore, vector fertility was not measured in the previous studies, and vector survival was only monitored for a limited period (7/8 days post-blood-feeding). The impact of the physiological difference between the various genera and species, however, cannot be ruled out [36]. These include the sensitivity of the vector’s midgut to proteases [54] as well as the volume of blood ingested by each vector species.

Another possible reason for the variability of these results could be the system that was used to make these recombinant vaccines. In previous immunisation studies, the *Pichia pastoris* expression system was used to express recombinant Akirin^*albopictus*^ and recombinant Q38 antigens [36–38], while this investigation used the *E. coli* expression system. The *E. coli* expression system has, however, been successfully used to express various recombinant proteins for the control of other arthropod studies [43]. One way to overcome this would be to standardize mosquito vaccine research, similar to that proposed for tick vaccines [55]. These parameters would need to include the standardisation of the expression system used, the type of adjuvant used, the experimental procedures followed to determine the antibody titres and vaccine efficacy, the sample size used to make these comparisons, as well the type and age of the experimental animals used within the study. This would allow for a more accurate comparison of results between different research groups.

When comparing the efficacy among the three recombinant vaccines, the difference was negligible. However, the chimeric nature of Q38 makes this antigen a valuable multi-target arthropod vaccine. Previously, the efficacy of Q38 was tested on *Ixodes ricinus*, *Dermacentor reticulastus* (ticks), and *Phlebotomus perniciosus* (sandflies) [38, 56]. In *P. perniciosus,* the ingestion of anti-Q38 antibodies significantly reduced vector oviposition by 16–26% [38]. The ingestion of anti-Q38 antibodies, by *I. ricinus,* significantly reduced vector longevity by 4.25-fold and moulting by 38%, while the ingestion of anti-Q38 antibodies significantly reduced the mass of *D. reticulastus* by 25% and moulting by 15% [56]. These previously reported findings, as well as those observed for the ingestion of anti-Q38 antibodies, which significantly reduced the reproductive capacities and vector survivorship of *Ae. albopictus* [38] and *An. arabiensis*, demonstrate the ability of using this recombinant vaccine to target multiple ectoparasitic infestations simultaneously using various agricultural blood hosts.

The conserved structure and function of Akirin throughout the metazoan [17, 25] have given rise to the question of safety when vaccinating vertebrates because of autoimmunity. As in previous experiments with different hosts, physiological or pathological alterations were not observed in vaccinated mice [14]. This suggests that, as expected, the antibody response was directed against non-self-epitopes, thus reducing the possibility of detrimental effects to the vaccinated host. Furthermore, Akirin is an intracellular antigen and, through still unknown mechanisms, the antibody can enter arthropod but not mammalian cells [8]. Additionally, effective immunisation of various hosts, for the control of numerous vector infestations, displays the low risk of inducing an autoimmune response in vertebrates [14, 36, 38, 43].

Challenging various blood hosts with whole protein or antigenic peptides may provide a valuable intervention against several ectoparasites including the untargeted exophagic and exophilic *An. arabiensis* mosquito populations. This vector control strategy could also impact secondary malaria vectors, or vectors of other diseases, if similar detrimental effects are caused within these vectors. Further studies, however, still need to be performed to determine if agricultural or domestic animals, vaccinated with recombinant Akirin vaccines, can elicit a similar response against *An. arabiensis* to those observed when recombinant Akirin vaccines were evaluated using balb/c mice. Furthermore, the half-life of these antibodies will need to be assessed within the reservoir hosts to determine how often booster vaccines will need to be administered to the agricultural or domestic animals.

This form of zooprophylaxis could therefore be applied in combination with other vector control strategies to divert *An. arabiensis* away from human hosts. Simultaneously, the *An.* arabiensis vector population would be targeted when feeding on these hosts by ingesting the anti-Akirin antibodies. This would cause a reduction of vector survivorship and reproductive capacities. By reducing *An. arabiensis* survivorship as well as reproductive fitness, a reduction in vector population abundance would ultimately occur. A reduction in vector abundance would have a significant impact on malaria transmission, especially in countries with low-level seasonal malaria transmission, such as South Africa.

## Conclusions

The results reported here were the first showing that hosts vaccinated with recombinant Akirin^*arabiensis*^ can develop a protective response against mosquito populations. Hosts vaccinated with recombinant Akirin^*albopictus*^ and recombinant Q38 also developed a protective response against *An. arabiensis*, and thus existing vaccines could be used to target this vector population. The reduction in *An. arabiensis* survival and reproductive capacities, due to revivor-host immunisation, has provided a step toward the development of a novel target for the control of *An. arabiensis.*

## Supplementary Information

**Additional file 1.** Data for survival analysis.

**Additional file 2.** Fecundity and fertility dataset.

**Additional file 3.** Calculation for vaccine efficacy.

**Additional file 4. **Overall vaccine efficacy.

## Data Availability

The datasets generated for this study are available as additional files.
