# Supplementary material for: Additional evidence on the efficacy of different Akirin vaccines assessed on Anopheles arabiensis (Diptera: Culicidae)
Source: Parasit Vectors. 2021 Apr 20;14:209. doi: 10.1186/s13071-021-04711-8 (PMC8056099; doi:10.1186/s13071-021-04711-8)
Supplement: Supplementary file 1 — Additional file 1. Data for survival analysis. [file 13071_2021_4711_MOESM1_ESM.docx]

**Additional File 1: SDS-PAGE and Western blot analysis**

The transformed cells and glycerol stocks were used to successfully express the respective recombinant proteins using the *E. coli* expression system. This was attested when the eluted fractions were analysed on the SDS-PAGE gel for the respective proteins. Due to the highly conserved nature of Akirin/Subolesin, all three recombinant proteins yielded similar results, and thus only the results from Akirin*^arabiensis^* were displayed below (Additional Figure 1). The recombinant proteins were observed at 30kDa on the SDS-PAGE gel (A), and was subsequently used for Western blot analysis (B). As seen in previously published literature, Akirin forms multimers and thus appears as multiple bands on the SDS-PAGE gel and Western blot [43]. This, however, does not affect the efficacy of the recombinant vaccines [36; 43]. When quantified, it was determined that 0.66mg of recombinant Akirin*^arabiensis^*, 0.45mg of recombinant Akirin*^albopictus^*, and 0.50mg of recombinant Q38 were expressed in total.


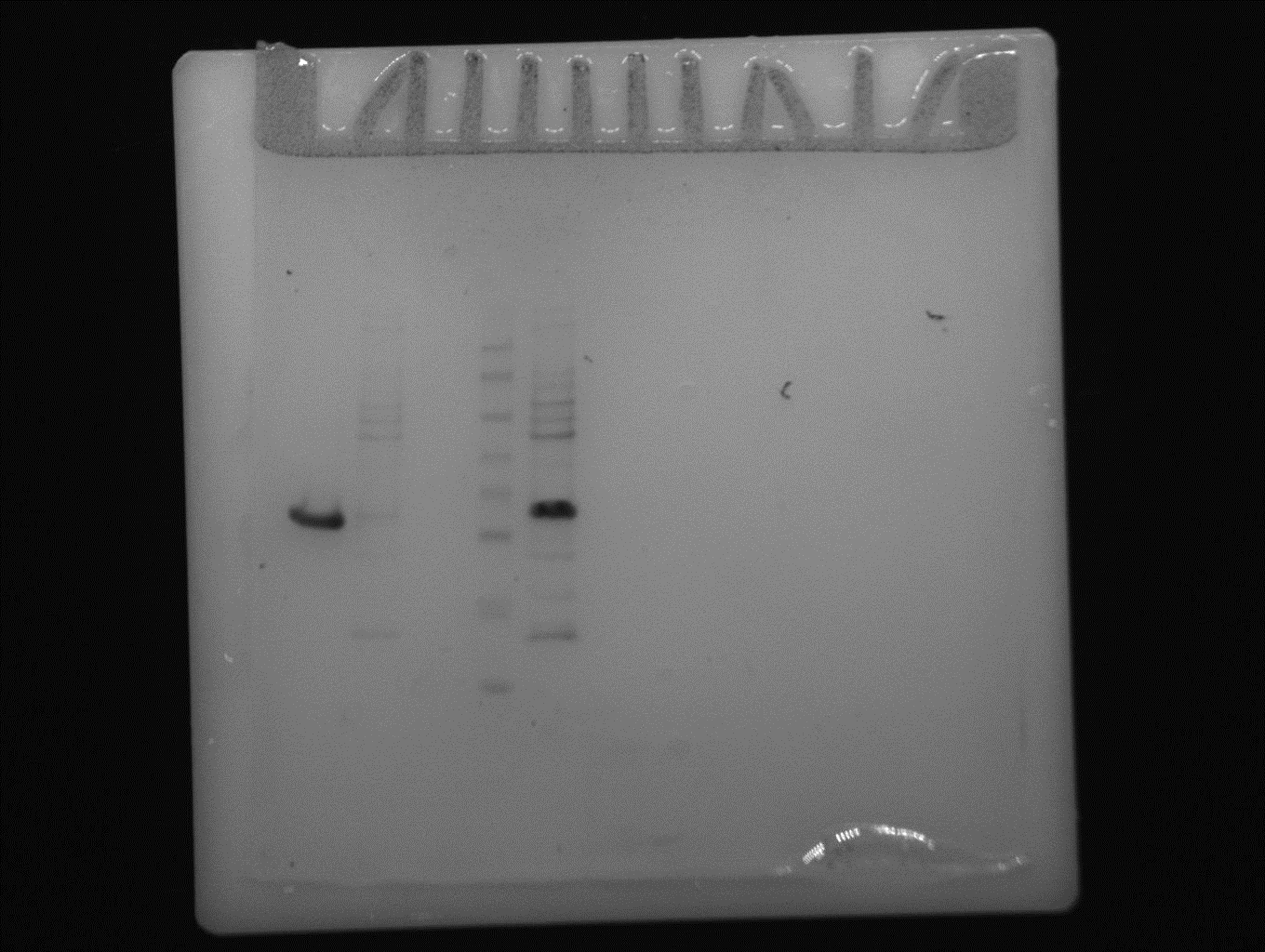


**A**

M 1

260

140

100

70

50

40

35

25

15

10

**kDa**


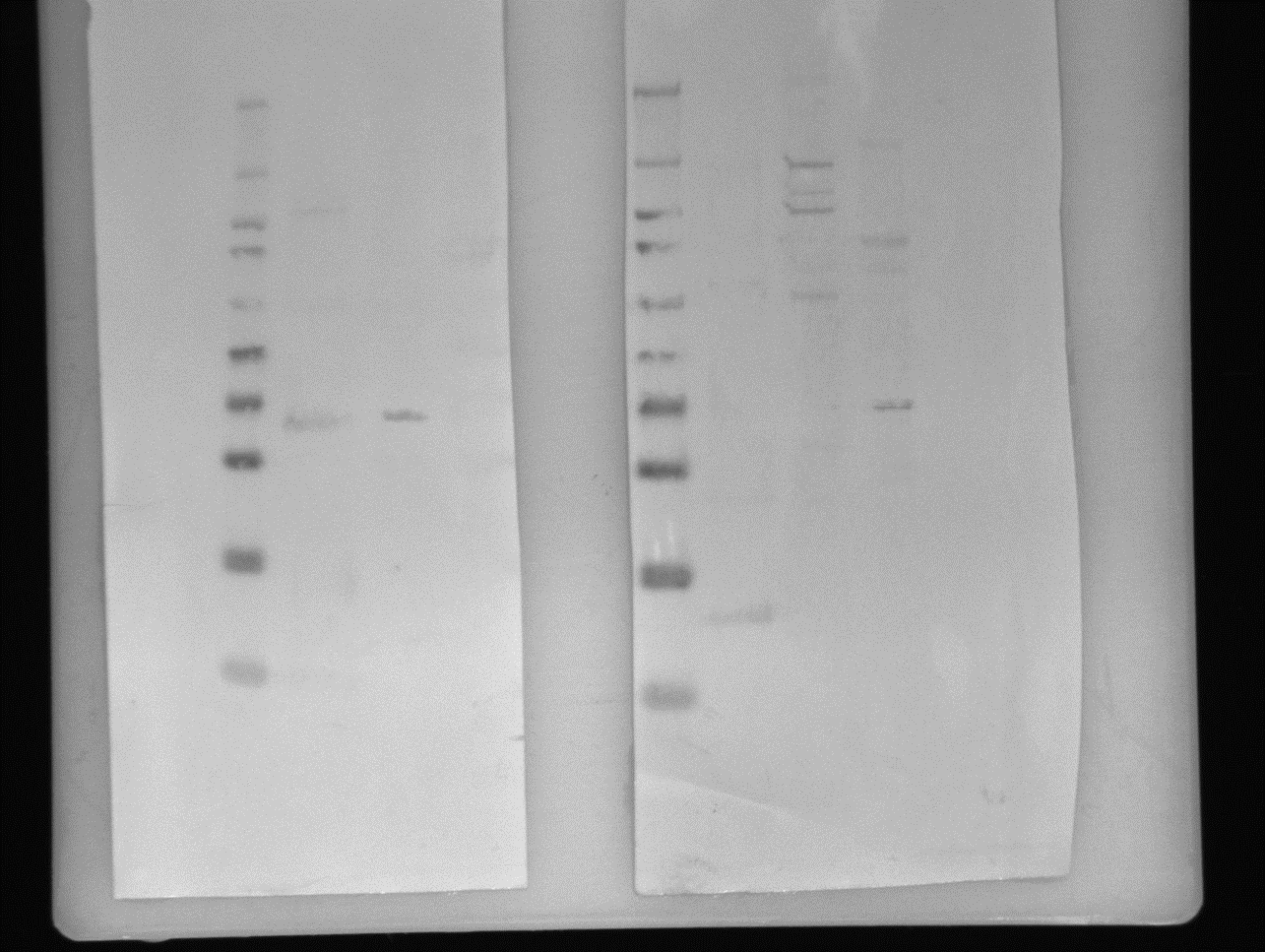


260

140

100

70

50

40

35

25

15

10

M 1

**B**

**kDa**

**Additional Figure 1.** The purified HIS-tag expressed Akirin*^arabiensis^* recombinant protein electrophoresed on a 12% SDS-PAGE precast gel (180V, 180mA) (A), and used for Western blot analysis (B). M: Spectra™ multicolor broad range protein ladder (ThermoFisher Scientific, 26634). Lane 1: eluted fraction containing the respective recombinant protein. Red dots denote the position of the recombinant antigen. Other protein bands in some of the samples correspond to *E. coli* contamination proteins, and aggregation or degradation products of the recombinant antigens [43].
